# Theoretical and practical development of the TOPSY self-management intervention for women who use a vaginal pessary for pelvic organ prolapse

**DOI:** 10.1186/s13063-022-06681-3

**Published:** 2022-09-05

**Authors:** Lucy Dwyer, Carol Bugge, Suzanne Hagen, Kirsteen Goodman, Wael Agur, Melanie Dembinsky, Margaret Graham, Karen Guerrero, Christine Hemming, Aethele Khunda, Doreen McClurg, Lynn Melone, Ranee Thakar, Rohna Kearney, The TOPSY Team

**Affiliations:** 1 The Warrell Unit, St. Mary’s Hospital, Manchester University Hospitals NHS Foundation Trust, Manchester Academic Health Science Centre, Manchester, UK; 2 Health Sciences & Sport, University of Stirling, Stirling, UK; 3 Nursing, Midwifery & Allied Health Professions Research Unit, Glasgow Caledonian University, Glasgow, UK; 4 NHS Ayrshire & Arran, Crosshouse Hospital, Kilmarnock, UK; 5 School of Medicine, Dentistry & Nursing, University of Glasgow, Glasgow, UK; 6 Dunlop, UK; 7 Department of Urogynaecology, NHS Greater Glasgow & Clyde, Glasgow, UK; 8 Aberdeen Maternity Hospital & Aberdeen Royal Infirmary, Grampian University Hospitals NHS Trust, Aberdeen, UK; 9 South Tees Hospitals NHS Foundation Trust, James Cook University Hospital, Middlesbrough, UK; 10 Croydon Health Services NHS Trust, Croydon University Hospital, Croydon, UK

**Keywords:** Prolapse, Pessary, Self-management, Self-efficacy, Randomised controlled trial (RCT), Intervention development

## Abstract

**Background:**

Pelvic organ prolapse (POP) is a common condition in women, where the downward descent of pelvic organs into the vagina causes symptoms which impacts quality of life. Vaginal pessaries offer an effective alternative to surgery for the management of POP. However, the need for regular follow-up can be burdensome for women and requires significant healthcare resources. The TOPSY study is a randomised controlled trial which aims to determine the clinical and cost-effectiveness of self-management of vaginal pessaries. This paper describes the theoretical and practical development of the self-management intervention.

**Methods:**

The intervention was developed using the MRC complex intervention framework, normalisation process theory (NPT) and self-management theory. The intervention aims to boost perceived self-efficacy in accordance with Bandura’s social cognitive theory and is guided by the tasks and skills Lorig and Hollman describe as necessary to self-manage a health condition.

**Results:**

The TOPSY intervention was designed to support women to undertake the medical management, role management and emotional management of their pessary. The six self-management skills described by Lorig and Hollman: problem-solving, decision-making, resource utilisation, formation of a patient-provider partnership role, action planning and self-tailoring, are discussed in detail, including how women were supported to achieve each task within the context of pessary self-management. The TOPSY intervention includes a self-management support session with a pessary practitioner trained in intervention delivery, a follow-up phone call 2 weeks later and ongoing telephone or face-to-face support as required by the woman initiated by contacting a member of the research team.

**Conclusions:**

The TOPSY study intervention was developed utilising the findings from a prior service development project, intervention development and self-efficacy theory, relevant literature, clinician experience and feedback from pessary using women and members of the public. In 2022, the findings of the TOPSY study will provide further evidence to inform this important aspect of pessary management.

**Trial registration:**

ISRCTN Registry ISRCTN62510577. Registered on June 10, 2017

## Background

Pessary self-management constitutes standard care at many healthcare organisations around the world [1–4], yet it is much less frequently recommended within UK pessary practice [5]. However, there is a lack of robust evidence to determine whether self-management offers benefits above and beyond clinician-led pessary care. Furthermore, there is a lack of guidance regarding how women should be supported to self-manage their pessary and the skills and knowledge required. The Treatment Of Prolapse with Self-care Pessary (TOPSY) study is an NIHR HTA-funded multicentre randomised controlled trial which aims to determine the clinical and cost-effectiveness of self-management of vaginal pessaries to treat pelvic organ prolapse [6, 7]. A power calculation was undertaken which determined that 330 women should be recruited and randomised, to ensure the ability to test for a significant difference in clinical outcome whilst allowing for attrition [7]. Recruitment took place across 21 centres in Scotland and England commenced in April 2018, and by February 2020, 340 women had been recruited. This manuscript details the theoretical and practical development of the self-management intervention.

## Pessary management

Pelvic organ prolapse (POP) is defined as the downward displacement of one or more of the pelvic organs including the uterus, vaginal compartments, bowel and bladder [8]. POP symptoms include seeing or feeling a vaginal bulge, a heaviness or a dragging sensation, difficulties voiding or defecating and sexual dysfunction, all of which can significantly negatively impact a woman’s quality of life [8].

A pessary is a medical device which can be inserted into the vagina to provide mechanical support to the prolapsed organs [9]. Pessary management of POP offers women comparable treatment outcomes to surgery in terms of reported symptoms and quality of life [10] and absence of bulges and no desire for further treatment [11]. A pessary may be a particularly desirable option for women who have not completed their family, are unfit for surgical management or would simply prefer to avoid the risks that POP surgery entails [12]. There are a wide variety of pessaries available, offering a conservative, long-term management option to women. In the UK, pessary follow-up is usually 3–6 months [13]. At each appointment, the pessary is removed, the vaginal tissues examined and either a new pessary or the same pessary replaced after cleaning. The need for regular follow-up is often cited as a reason why women opt for surgical management of POP due to the inconvenience of frequent appointments [14–16]. Moreover, clinician management means women are denied autonomy in how and when to use their pessary. The cost of regular pessary follow-up appointments is significant, with over 86,000 pessaries inserted annually in English NHS services alone [17].

## Pessary self-management

Self-management involves an individual and healthcare professional working collaboratively with or without wider social support to maximise health [18, 19]. This is achieved by facilitating the individual to participate in healthcare regimes, the individual engaging in health protection and promotion activities, the individual monitoring and managing symptoms and the subsequent impact on daily activities, emotions and relationships and the creation of a new, meaningful life role [18].

To translate this to pessary care, pessary self-management can be defined as the pessary practitioner and woman working collaboratively to facilitate the woman to use her pessary in a way that best meets the needs of her everyday life and minimises the impact of her POP and pessary upon emotions and relationships. There is evidence which confirms the feasibility of women removing and inserting their own pessary [16, 20, 21]. These activities can be defined as self-care skills. However, without additional education and support to empower the woman to make active decisions regarding pessary management of her POP, it is arguable the woman is not self-managing her condition. To meet the definition of self-management, the woman must be an active participant in her care. Therefore, a woman who has her pessary changed by a clinician at regular follow-up appointments and does not undertake activities to manage or maintain the pessary in the interim would not be classed as self-managing her pessary. However, a woman can be classed as self-managing her pessary whether she removes or inserts her pessary daily, weekly or annually. The frequency of the activity is not important; it is the responsibility, engagement and ability to resolve issues that occur that matter.

There is limited evidence about the effectiveness of pessary self-management [22], but there is a widespread commentary about the use of self-management in pessary services. A service improvement project provided some information regarding UK pessary users and their willingness to self-manage [14]. In total, 88 women agreed to be taught how to self-manage their pessary. Each woman had a 45-min appointment with a specialist women’s health physiotherapist who provided education on how to insert and remove a pessary and then supervised the woman whilst practising this. Women were also provided with a written information sheet and access to an online video. Follow-up phone calls to offer ongoing support were arranged between the physiotherapist and woman 2 weeks, 1 month, 3 months and 6 months later. At 6 months of follow-up, 63 women (73%) were continuing to self-manage their pessary. Self-managing women reported improved convenience, comfort and access to help and support. Ninety-seven per cent of women self-managing at 6 months planned to continue with long-term pessary management of their POP compared with 70% in the clinician-led care group. The success of this project and the feedback provided by stakeholders regarding the self-management intervention provided the basis for the Treatment Of Prolapse with Self-care Pessary (TOPSY) study self-management intervention. TOPSY is an NIHR HTA-funded multicentre randomised controlled trial to determine the clinical and cost-effectiveness of self-management of vaginal pessaries to treat pelvic organ prolapse [7].

At present, there is no rigorous evidence regarding the required constituents of pessary self-management support. Therefore, clinical grant co-applicants for the TOPSY study were asked to review the proposed intervention and information materials to ensure the necessary components were included. The proposed intervention including patient information was reviewed by the Royal College of Obstetricians and Gynaecologists (RCOG) Women’s Voices panel, an online group of over 600 members of the public who have experience using women’s health services, in addition to the pessary using women who were grant co-applicants. Amendments to the intervention and associated documents based on lay feedback were made including adding more illustrations, introducing further details about pessary insertion and removal. The activities undertaken with a variety of stakeholders and the clinician-led input into the intervention ensured it was coherent to potential participants and clinicians delivering the intervention [23].

## Intervention development

The TOPSY intervention focuses on the self-management of vaginal pessaries. To develop the intervention, the research team considered intervention development guidance to create a programme theory [24]. The TOPSY intervention was developed prior to the publication of the draft updated complex intervention guidance [25] and was therefore based on the 2008 guidance [24]. To present the paper using contemporary language, we have however drawn upon the language used in the new draft guidance [25, 26].

The 2008 model of intervention development outlines three key areas:

- Identifying the evidence base
- Identifying or developing theory
- Modelling process and outcomes

In order to establish the evidence base, a literature search was undertaken in 2013 with the aim of identifying clinical trials exploring pessary self-management. No trials were identified. However, one paper outlining a self-management intervention was found [14]. The evidence base to underpin the intervention was therefore difficult to identify. A lack of robust evidence regarding pessary self-management was also found by the team undertaking a Cochrane review of pessaries for managing pelvic organ prolapse in 2020 and 2013 [27, 28]. Due to the lack of rigorous evidence to inform the intervention, informal consensus development methods were followed [29]. Both clinical co-applicants, including medical doctors, nurses, and physiotherapists, and pessary-user co-applicants on the grant worked together to achieve consensus on the proposed intervention. A draft protocol for pessary self-management support was created by two clinician co-applicants (LD, RK) drawing upon the sole paper identified during the literature search [14] and their own clinical practice. This was subsequently reviewed by both clinical and pessary user co-applicants. Feedback was received and reviewed by the co-applicants, and changes were made accordingly. In instances where there was discordance, the group discussed the area of disagreement and overall consensus guided whether or not a change was made. One example of this was whether it was necessary for a woman to be informed about the correct positioning of her pessary using medical terminology. Whilst it was acknowledged informing women about the anatomy of prolapse was beneficial, there were concerns advising women a pessary had to be positioned in an exact way may overcomplicate and cause additional anxiety for women. Moreover, the clinicians concurred that not all pessaries can be positioned as cited within the literature; therefore, a pragmatic approach to positioning would be preferable. As a result, it was agreed women would be informed as long as the pessary was comfortable and supported the prolapse, it could be deemed as correctly positioned. Once developed, the self-management support documents were reviewed by the RCOG Women’s Voices panel, following which further amendments were made to ensure the contents offered pragmatic and realistic self-management advice that met women’s information needs.

## Normalisation process theory

The intervention was developed using the underlying concepts of the normalisation process theory (NPT) framework [23]. The NPT framework highlights the factors needed for the successful implementation and integration (normalisation) of interventions into the real world [23]. The main components of NPT are coherence, cognitive participation, collective action and reflexive monitoring [23]. Consideration of these components ensures an intervention is coherent, is designed to ensure stakeholders will be engaged in intervention delivery, takes current working practices into consideration and is informed by stakeholder feedback [23]. Whilst describing the theoretical and practical development of the intervention, the NPT framework will be used to structure the details of the processes undertaken to design an intervention for TOPSY which could be feasibly incorporated into everyday practice.

## Underpinning self-management theory

The foundation of our programme theory is self-management theory which in turn draws upon self-efficacy theory—each of these is covered in more detail below. To underpin our programme theory, we developed a logic model that demonstrated the links between the self-management intervention, and hence self-management theory, self-efficacy and quality of life.

Bandura [30] defined self-efficacy as an individual’s beliefs about their capabilities to produce designated levels of performance that exercise influence over events that affect their life [23]. Self-efficacy is important because it determines how individuals feel, think and motivate themselves and how they behave [30]. In terms of health, having a strong sense of self-efficacy motivates an individual to set and meet challenging goals, overcoming obstacles that may arise [30]. Therefore, in order to successfully facilitate an individual to self-manage their condition, it is essential the self-management programme incorporates methods to boost an individual’s perception of self-efficacy to ensure they have the resilience and confidence to utilise the skills and knowledge they have obtained, adapting them as necessary [18].

Lorig and Holman [19] describe three tasks and six skills they believe to be necessary to self-manage a condition. The tasks specified are medical management of the condition, role management and emotional management. The necessary skills are problem-solving, decision-making, resource utilisation, formation of patient-provider partnership role, action planning and self-tailoring. The TOPSY intervention (Appendix) was modelled upon these tasks and skills with the aim of better supporting women who wished to self-manage their pessary to overcome potential barriers.

### Medical management

Medical management is about how a person medically manages their health condition. In TOPSY, women were supported to have the skills to medically manage their POP by being taught pessary self-care. To facilitate this, women were given a 30-min support appointment and a 2-week follow-up call. During the support appointment with a healthcare professional, they were provided with information about POP including relevant anatomy and management options with specific emphasis on pessary management including how a pessary works. Women were also provided with an example pessary to handle so they could practice manipulating the pessary as necessary to insert and remove it. Once comfortable with handling and compressing the pessary, the woman was encouraged to practise removing and inserting it. Whilst there is no specific evidence to inform practice in pessary self-management support, there is literature about how to support the effective learning of other similar skills such as intermittent self-catheterisation (ISC). Individuals taught ISC emphasised the importance of effective communication from the healthcare professional, good interpersonal skills to provide reassurance and overcome embarrassment, a private environment, both written and oral information and at least one practical demonstration, with some participants stating a preference for more than one practical demonstration [31]. The findings of another qualitative study exploring the experience of being taught ISC demonstrate that performing the skill under supervision to be determined as competent resulted in women feeling under pressure to ‘pass’ a test [32]. This demonstrates the importance of an individualised approach to supporting the learning of a new healthcare skill, in this instance, pessary self-management. In view of this, as part of the TOPSY intervention, women were supported to practice repeated insertion and removal of the pessary under the supervision of the healthcare professional providing the support. However, should the woman be unable or not feel sufficiently comfortable to practice this skill during the appointment, she was encouraged to do this at home instead, and this was followed up during the 2-week follow-up phone call. All women who were randomised into the self-management group and received the intervention accordingly were contacted by telephone after 2 weeks to check whether they had been able to remove and insert the pessary independently at home and whether they had experienced any difficulties or required further support. Complications such as discharge, discomfort or pain and bleeding are common amongst pessary users [33]. However, when supported to manage these issues, this need not result in pessary discontinuation [33]. Details regarding how we facilitated women to problem solve are reported below.

### Role management

There may be a complex relationship between a healthcare professional and patients learning to self-manage their condition [34]. Patients may be used to deferring to the healthcare professional providing their care, due to their academic expertise within the area, despite this minimising the value of the patient’s lived experience of their condition [34]. To suddenly change the relationship and ask a patient to take responsibility for their own care is argued to require sensitivity and support to ensure the patient is not confused about the abrupt change in the power dynamics of clinician and patient roles [34]. Lawn et al. [34] suggest a patient’s role in self-management is undertaking and being engaged in self-management activities whilst also accepting support from healthcare professionals as required. Therefore, the healthcare professional and patient share responsibility for the management of the condition [34]. In this instance of pessary self-management, women were encouraged to take ownership of their role in managing their pessary. Rather than clinician-led follow-up at pre-determined intervals, this was instead guided by the woman depending on her needs. It is helpful to emphasise the value of the woman’s experience as a pessary user and the benefits this offers during pessary insertion and removal. For example, whilst determining the correct size and type of pessary requires clinical knowledge and experience [35], the process of removing and inserting a pessary does not. As the woman inserting her own pessary is able to feel whether the pessary is positioned comfortably and effectively to reduce the prolapse, this is a pragmatic benefit a healthcare professional inserting a woman’s pessary does not have. This demonstrates the valuable contribution that both the healthcare professional and woman bring to the pessary self-management relationship. Following the intervention, women should feel empowered with the additional knowledge and confidence provided to undertake the self-management role, whilst still feeling supported by a healthcare professional in case of any concerns or issues.

### Emotional management

POP significantly affects a woman’s emotional well-being [36]. Furthermore, the treatment of POP can have either a positive or negative impact on emotions [36]. Therefore, women being taught to self-manage their pessary may feel positive about the process as their pessary is working well, but they desire more autonomy in how they use their pessary. Conversely, other women may have negative emotions about the process as they have had a recurrence of POP following surgery, wanted surgical management but were unsuitable or are having to learn to self-manage their pessary because it is continually expelled and therefore will need to be reinserted frequently by the woman. Taking the range of emotions women may be experiencing into consideration may facilitate a healthcare professional to better meet her needs [36]. To facilitate the woman to overcome the negative emotions she may experience related to living with POP or self-managing her pessary, the healthcare professional reinforced the benefits of pessary self-management and spoke positively about pessaries as an effective POP management option which may not be inferior to surgery [10].

At present, there is a lack of evidence exploring the barriers to pessary self-management. However, an exploratory study of the barriers to the use of vaginal dilators identified women had concerns of pushing the dilator in too far, hurting themselves or breaking the dilator [37]. It is acknowledged there are differences in the use of vaginal pessaries and dilators; however, consensus between clinical co-applicants suggested the fears women have about using vaginal dilators are shared by many women learning how to self-manage their pessary. Therefore, the healthcare professional also discussed common concerns women using or self-managing pessaries may have, for example, the risk of infection, or that a pessary could be inserted incorrectly. The ability to self-manage a pessary means that if desired, a woman can remove her pessary to be sexually active. Meriwether et al. [38] reported 70% of sexually active pessary using women regularly removed their pessary before sexual activity, usually due to concerns expressed by their partner. Therefore, this is clearly an important aspect of pessary self-management for women who are, or wish to be, sexually active. A discussion about how POP or pessary management has affected the woman’s sexual function and the emotional impact of this is indicated. The woman was also given the opportunity to ask any questions they may have or express if anything was worrying them.

### Problem-solving

Hill-Briggs devised an applied model of problem-solving in chronic illness self-management which details four key components [39]. These are disease-specific knowledge, transfer of past experience, problem-solving skills and process and problem-solving orientation [39]. In relation to pessary self-management, women were supported to problem solve by ensuring they had sufficient disease-specific knowledge through the provision of written and verbal information including POP, pelvic anatomy and how a pessary works. The woman’s prior experience of pessary use could be used to demonstrate effective self-management problem-solving by ensuring women understand potential causes for problems and appropriate responses [39]. For example, if a woman previously sought assistance from a healthcare professional for reinsertion of a pessary due to pessary expulsion caused by excessive straining, the woman can be encouraged to instead reinsert the pessary herself and take measures to avoid excessive straining in future, as this prior experience makes the problem-solving behaviour more relatable. Ensuring a woman had a positive problem-solving orientation was achieved by ensuring the woman has sufficient self-efficacy to feel confident enough to resolve unexpected issues or to seek clinician support if necessary. The intervention therefore aimed to ensure women felt self-efficacious and viewed pessary self-management as a solvable challenge through positive reinforcement and encouragement during the session.

### Decision-making

To facilitate day-to-day decision-making managing a chronic condition, individuals require sufficient and relevant information [19]. Providing women with an understanding of potential issues and when to seek clinician advice or assistance if necessary, enables women to make decisions about how to deal with any concerns or problems with the pessary knowing there is support at hand if required. For example, if informed vaginal discharge is a common side effect for women using pessaries, whether self-managing or not, they may be less likely to be concerned if they experience non-bothersome discharge and instead decide to remove and wash the pessary more frequently.

### Resource utilisation

As with other types of patient education [19], Murray et al. [40] found the provision of an information brochure in addition to verbal information, compared to verbal information alone, for women being taught to self-manage their pessary resulted in women being more satisfied, confident and knowledgeable. With this in mind, the TOPSY intervention included the provision of an information sheet reiterating the self-management information given verbally, so it could be referred to at a later point as desired. Women were also made aware of an online video about pessary self-management created utilising feedback from pessary users for Kearney and Brown [14] service improvement project. To ensure consistency in the information about POP provided to women being taught self-management, it was agreed a copy of the International Urogynaecological Association (IUGA) POP patient information sheet (https://www.yourpelvicfloor.org/media/Pelvic_Organ_Prolapse_RV3.pdf ) be given and utilised during the self-management training. This information sheet includes topics such as the anatomy and physiology of POP, the causes of POP and POP management options. To avoid the suggestion any differences in outcome measures between the two groups could be explained by increased knowledge and understanding of POP, it was agreed all participating women should be provided with the information sheet upon randomisation.

### Formation of a patient-provider partnership

In order to facilitate individuals to take responsibility for their condition, it is necessary to ensure they feel in control of their health, rather than simply adhering to their clinician’s instructions [19]. The intervention was designed to encourage women to view the healthcare professional providing self-management training as an equal partner aiming to facilitate best possible pessary care rather than a clinician making decisions about the woman. This was achieved by empowering women with information to ensure they felt sufficiently informed to ask questions and make decisions about their POP and pessary care. Moreover, the intervention was designed to be as pragmatic and unmedicalised as possible. For example, as previously discussed, instead of specifying how to position the pessary in terms of a complex anatomical description, women were advised the pessary was positioned correctly if it was comfortable and the POP was managed.

### Action planning

Lorig and Hollman advocate setting individuals a short-term achievable self-management goal to boost self-efficacy [19]. Analysis of action plans suggests they increase feelings of self-efficacy and the likelihood of completing the action plan after 6 months [41]. An action plan should be specific, important to the individual, public and short-term [41]. Lorig et al. [41] also advocate an individual scoring their confidence in successfully completing the action plan to ensure additional targeted support if necessary. For the TOPSY study, the skill to be mastered was independently removing and inserting a pessary. This was therefore specific, important, and public-in terms of being known by the healthcare professional rather than solely the patient and short term as women were asked to practice this skill over the following 2 weeks. After 2 weeks, the healthcare professional arranged to call to answer any questions or concerns the woman might have. After this, women were encouraged to remove their pessary whenever they wished but at least every 6 months.

### Self-tailoring

Self-tailoring describes changes made by the individual based on principles they have learnt and self-management skills [19]. Whilst yet unproven, a perceived benefit of pessary self-management is the woman’s ability to make autonomous decisions about how and when to use their pessary [22]. Despite this, a number of publications detailing pessary self-management protocols specify a frequency of pessary removal for the woman to follow [20, 42, 43]. Due to the lack of evidence about the required frequency of pessary removal for self-managing women, it was decided women receiving the TOPSY intervention be advised to remove their pessary at least 6 months [7]. This mirrors the frequency of removal for women receiving clinician-led follow-up in accordance with the NICE guidelines [44]. However, women were encouraged to self-tailor pessary use other than this, removing the pessary as frequently or irregularly as they wished. It was also explained to women that with clinic-based care, women attend a follow-up appointment at a set interval depending upon the agreed schedule at that organisation, typically 3–6 months. For women who were self-managing their pessary, no follow-up appointment was arranged prior to the end of the study at 18 months. However, women could request follow-up at any point and as frequently as they wanted, prior to that. This meant that where they were required, follow-up appointments met the individual needs of the women rather than a local protocol.

### Intervention delivery and training

Many different healthcare professional groups provide pessary care in the UK including nurses, medical doctors, physiotherapists, midwifes and clinical support workers [45]. Furthermore, there is extensive variation in training standards for pessary practitioners at different organisations in the UK [45]. This meant a pragmatic approach regarding who delivered the intervention was necessary. In addition to strengthening collective action by ensuring a multidisciplinary approach to intervention delivery which was compatible with existing work practises [23], the findings can also be generalised to a range of healthcare professionals, rather than applicable solely to a specific professional group. It was specified the TOPSY self-management intervention should only be delivered by pessary practitioners already trained to the local specifications at their organisation and providing pessary care as part of their clinical role. Prior to delivering the self-management intervention, pessary practitioners received intervention delivery training provided by a clinical member of the TOPSY team at a site visit. This presentation covered pessary self-management, each aspect of the intervention, why it was necessary and the information to be included. A training manual to refer back to if necessary was also provided. During the site visit, the TOPSY team ensured that the intervention was compatible with how pessary self-management was currently taught and could be feasibly delivered. By ensuring additional training was not onerous and did not conflict with established working, this ensured cognitive participation and collective action amongst clinicians and key stakeholders in intervention delivery [23]. Following the site visit, healthcare professionals who accepted delegated responsibility for intervention delivery were asked to sign a record confirming they had received training and felt confident to deliver self-management support as part of the trial.

A case report form was designed to record the delivery of each aspect of the intervention. This ensured standardised delivery of the intervention but also enabled reflexive monitoring [23]. Analysis of the case report form facilitated the identification of any recurring aspects of the intervention which clinicians did not deliver and the reasons for this. There were also free text boxes for clinicians to record whether they omitted or added any content to the intervention session. This enables analysis of how clinicians feel about the delivery of the intervention and for adaptions and improvements to be made if advocated. In addition to the analysis of the intervention delivery case report form, a sample of pessary using women and health care professionals were interviewed and a number of self-management support sessions and follow-up calls audio recorded as part of the process evaluation [6].

## Discussion

The limited evidence available suggests self-managing a pessary may offer benefits such as improved satisfaction, quality of life and increase the length of time a woman has pessary management for, without increasing the complication rates [22]. There may also be a cost-saving benefit if the number of clinician-led follow-up appointments can be reduced for women who are self-managing their pessary. TOPSY is a multicentre randomised controlled, designed to provide rigorous evidence about the clinical and cost-effectiveness of pessary self-management [7].

**Fig. 1:**
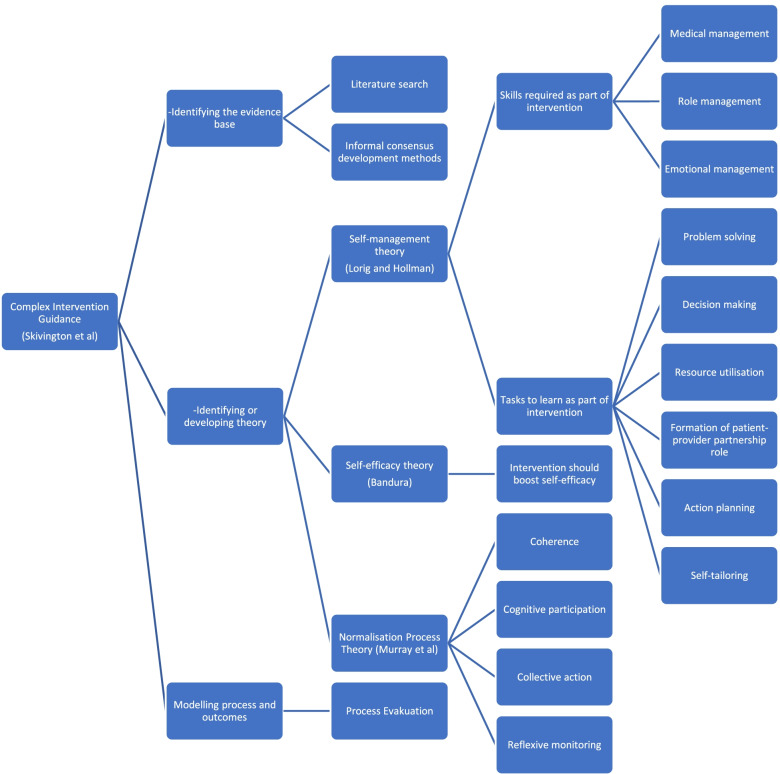
TOPSY intervention development diagram

Despite pessary self-management being frequently offered to pessary-using women around the world [1–4], there is lacking detail about the support women require to self-manage. This paper describes the theoretical underpinning behind the development of the TOPSY self-management intervention including complex intervention development theory [24], normalisation process theory [23] and self-management theories [19, 30, 39] (Fig. 1). Due to the lack of rigorous evidence to inform the clinical components of the intervention, it was designed pragmatically based upon the limited evidence available, clinician consensus and input from pessary using women.

## Conclusions

This paper describes the theoretical underpinning and practical development of the pessary self-management intervention for the TOPSY study. The TOPSY study intervention builds upon findings from a prior service development project, intervention development and self-efficacy theory, relevant literature, clinician experience and feedback from pessary using women and members of the public. In 2022, the findings of the TOPSY study, the largest UK trial exploring pessary self-management to date, will provide further evidence to inform this important aspect of pessary management.

## Data Availability

All data requests should be submitted to the corresponding author for consideration. Access to anonymised data may be granted following the review.

## Appendix

Information giving:

- Provide information on pelvic organ prolapse
- Provide information about pessary self-management
- Talk to the woman about taking care of her own health
- Talk to the woman about the potential benefits of pessary self-management
- Discuss managing emotions such as fear or anxiety related to pelvic organ prolapse, pessary management or pessary self-management
- Discuss the anatomy of the vagina and pelvic organs
- Talk to the woman about the type of pessary she uses and how it works

Practical demonstration:

- Provide the woman with a pessary to handle
- Demonstrate how to apply lubrication
- Demonstrate insertion of a pessary
- Demonstrate positioning of pessary
- Demonstrate removal of pessary
- Demonstrate cleaning of pessary
- Discuss storage of pessary when not in use
- Provide information about how to receive a replacement pessary
- Provide information about the discontinuation of pessary use if the woman becomes pregnant
- Discuss what to do in case of problems
- Give information about additional resources available (support phone number, link to video)
- Talk to the woman about common issues such as discharge or what to do when menstruating (if appropriate)

Practice pessary self-management:

- Facilitate the woman to practice pessary removal offering support and advice as required
- Facilitate the woman to practice pessary insertion offering support and advice as required

Action planning:

- Ask the woman to remove, clean and reinsert pessary at least once over the next 2 weeks
- Discuss with the woman after this she can remove/reinsert the pessary to accommodate her lifestyle
- Check if the woman has any further questions
- Make the woman aware of telephone support
